# Long-term outcome of in-patients with substance use disorders: A study from North India

**DOI:** 10.4103/0019-5545.44750

**Published:** 2008

**Authors:** Shubh M. Singh, Surendra K. Mattoo, Alakananda Dutt, Kaustav Chakrabarti, Naresh Nebhinani, Suresh Kumar, Debasish Basu

**Affiliations:** Department of Psychiatry, Postgraduate Institute of Medical Education and Research, Chandigarh - 160 012, India

**Keywords:** In-patient, outcome, predictor, substance use

## Abstract

**Background:**

Research into substance use disorders (SUD) has been unable to unequivocally demonstrate effectiveness of treatment modalities.

**Aims:**

The aim of the study was to study the long-term outcome after in-patient treatment in a cohort of patients admitted for SUD in a deaddiction unit of a hospital in North India.

**Materials and Methods:**

The case notes of all in-patients with a primary diagnosis of alcohol and/or opioid dependence syndrome (F10.24 and F11.24) in the calendar year 2006 were examined. All patients without any physical or mental comorbidity other than comorbid SUD were included in the study. They were contacted telephonically or their case notes examined in September, 2007. Status regarding abstinence or relapse was determined and data was analyzed.

Independent samples *t*-test and chi-square test were used for determining significance of difference between continuous and categorical variables respectively. Kaplan-Meier analysis was applied to find the survival times of different groups with the duration to relapse as variable of interest. Log rank test was applied to find the significance of differences in various groups. Cox's Regression analysis was applied to find the hazard ratio.

**Results and Conclusions:**

Data for 59.22% of patients included in the study were available for analysis. Mean survival time was 36.35 weeks (28.74-43.95, 95% CI) for patients across different groups, 36.71 weeks (26.24-47.18, 95% CI) for the alcohol group, 34.00 weeks (8.37-59.36, 95% CI) for natural opioids group, 37.53 weeks (26.33-48.73, 95% CI) for semi/synthetic opioids group and 17.00 weeks (3.39-30.60, 95% CI) for the mixed group. Kaplan-Meier analysis revealed that those who were following-up at time of evaluation had significantly longer durations to relapse. Deaddiction services should stress on keeping patients on follow-up as a means to better outcomes.

## INTRODUCTION

Substance use disorders (SUD) are chronic relapsing conditions,[1] with generally poor outcomes.[2–4] In fact, benefit of treatment vs. no-treatment in SUD is not firmly proven in either out-patient or in-patient settings.[5,6] Pharamacoprophylactic agents such as Naltrexone (NTX) and Disulfiram (DSF) in conjunction with non-pharmacological methods of treatment remain a major method of ensuring abstinence after detoxification but the effectiveness of these drugs remains doubtful as well,[7–9] and a combination approach of prophylactic drugs and psychosocial methods in the treatment of SUD has been mooted.[10] It is estimated that about 80% of all patients entering treatment for SUD have other comorbidities and these are associated with worse outcomes.[11,12] It is evident that the treatment outcomes in SUD are nebulous and are often confounded by comorbid conditions in patients. The data from India regarding effectiveness of treatment modalities in SUD is meagre and conflicting.[13–16] Therefore, the present study was designed to find the long-term outcome among patients without confounding physical or psychiatric comorbidities who had received in-patient treatment for SUD at a deaddiction center in India.

### Objectives

The objective of this study was to find the outcome in a cohort of patients who had received in-patient treatment for SUD in a deaddiction center in North India at 8-20 months after the index admission. The outcome measure of interest was continued abstinence or relapse as defined for the purpose of this study. Factors influencing the outcome were to be assessed and inferences drawn.

## MATERIALS AND METHODS

### Setting

The study was based at the 20-bedded Drug Deaddiction and Treatment Center (DDTC), Department of Psychiatry, Post Graduate Institute of Medical Education and Research (PGIMER), Chandigarh, India. The PGIMER is a multi-specialty teaching hospital located in North India and caters to a rural and urban population of about 40 million. The DDTC provides comprehensive out- and in-patient services for the substance abusers through a team of psychiatrists, clinical psychologists, psychiatric social workers (PSW), nurses, and a yoga therapist. Annually it caters to 400 or so new outpatients, 5000 or so follow up visits, and 250 or so in-patients. The profile of substances of abuse for which treatment is sought is: 40-60% alcohol, 40-60 % opioids, and 1-2 % each solvents, sedatives, tobacco, and cannabis. About 60% of patients also report tobacco dependence (about one-third each by smoking, chewing, and both), but they generally do not seek treatment for the same.

### Study population

The study population comprised of all the DDTC in-patients with a primary diagnosis of alcohol or opioid dependence syndrome (F10.24 or F11.24) as per the ICD-10[17] in the calendar year of 2006. Patients with a comorbid diagnosis of harmful use (F1x.1) or dependence syndrome (F1x.2) for other substances were also included as long as the main substance of abuse was alcohol or opioids. To control for the confounding effects of comorbid conditions as reviewed above, patients who had been diagnosed to be suffering from any major physical or psychiatric comorbidity, whether or not substance-related were excluded. These included disorders such as complicated withdrawal to any substance, substance induced psychotic disorders, cirrhosis of the liver, seizures or other such physical or mental disorders.

### Method

In-patient case notes for the year of 2006 were examined and patients fulfilling the inclusion criteria were identified. In September 2007, all of these patients were contacted by telephone. Wherever contact was possible, a verbal consent was obtained from the patient or family member. Thereafter, their current status as regards abstinence or relapse was asked for from either the patient or any other family member who could be contacted and was aware of these details. If telephonic contact was not possible, case notes of patients were examined. If the patient had followed up within the preceding month, the status as recorded at that visit was taken as the outcome. Consent was not obtained from the patients where outcome was obtained from the case notes. All those patients who could either be contacted telephonically or whose case notes were available as defined above were included in final analysis. If the case notes revealed that a patient to have not followed-up in the month prior to the assessment or if the patient was not accessible by telephone, then such a patient was excluded from the analysis. Thus, the time period of follow-up in patients included in final analysis ranged from 8 months to 20 months.

### Operational definitions

If the data were obtained telephonically, relapse was defined whenever the patient or family member reported any 3 or more of the following: that there was a strong desire to take the substance, that there was difficulty in controlling onset or termination or level of use, the typical withdrawal symptoms of that substance were being observed, there was increasing requirement as regards dose, there was neglect in performing other alternative activities and there was continued use despite harm. Abstinence was defined as no known use of substance that necessitated admission. Occasional use or use not amounting to dependence did not classify as relapse. We also made enquiries regarding substitution of drug with another. Relapse or abstinent status was defined as mentioned in the case notes in patients where telephonic contact was not possible.

### Variables of interest and statistical methods

Socio-demographic data were recorded using the profile sheet developed for this purpose in the department. Clinical variables recorded included the main diagnostic groups as regards the preferred substances of intake. These groups were alcohol, natural opioids [opium and poppy husk], and synthetic/semi-synthetic opioids [heroin, dextropropoxyphene (DPP) capsules, buprenorphine and pentazocine (intravenous injections), codeine containing cough syrups (CCCS), and diphenoxylate tablets], and mixed (where both F10.24 and F11.24 were diagnosed). Comorbid dependence or harmful use syndromes such as that with nicotine (F17.24) or cannabis (F12.24) or sedatives (F13.24) were noted but not entered into the analysis separately. These patients were included in the study as has been mentioned earlier. The outcome at discharge was taken as improved wherever mentioned as such in the case notes. This would imply that the patient had been discharged after satisfactory detoxification and institution of pharamacoprophylactic agents and other non-pharmacological means of deaddiction. The outcome was taken as not improved whenever the patient was recorded to have ‘absconded from the ward’, ‘discharged on disciplinary grounds’, ‘left the ward against medical advice’, or ‘not complied with pharmacoprophylaxis institution and non-pharmacological means of deaddiction’. The pharamacoprophylactic medication was noted. These included Disulfiram (DSF), Naltrexone (NTX), DSF+NTX or none. Finally, patients were considered to be following-up if case notes revealed that they had followed-up in the month prior to the date of assessment. Chi-Square test was used to find differences between groups for categorical variables. For continuous variables, an independent sample *t*-test was used. Kaplan-Meier survival curves were generated with the duration to relapse as the survival time variable and abstinence or relapsed status as the survival status variable. The variables of diagnostic groups, outcome at discharge, pharmacoprophylaxis and follow-up were separately entered as factors in the above analysis and the Log-rank test was used to quantify the differences in the survival in each of these analyses. The variables for which the difference was found to be statistically significant (*P* ` 0.05) were entered in a Cox's regression analysis to compute the hazard ratio with 95% confidence interval. All statistical analyses were done with the SPSS version 10.0 statistics software for windows (SPSS Inc., Chicago, IL).[18]

## RESULTS

A total of 246 patients were admitted to the DDTC ward in the year 2006. A majority of patients were admitted with a primary dependence on synthetic/semi-synthetic opioids followed by alcohol. Of the 246 cases, 103 fulfilled the inclusion criteria for entry into the study. We were able to contact telephonically 40 patients and identify records of 21 patients who were following-up as defined earlier (*N*=61, 59.22%). Of the 40 patients contacted telephonically, 10 (25%) claimed to be abstinent. Among the 21 patients who were following-up and whose records were available, 15 (79.37%) were abstinent as per records on the last follow-up (*N*=25, 40.98%). Table 1 shows the diagnostic break-up of the patients who could be contacted or whose records were available and those who could not be contacted. There was no significant difference as regards the age of patients and the main substance of abuse. Those patients with synthetic/semi-synthetic opioid dependence who could be contacted were younger than those who could not be contacted but this difference was not significant. There was also no significant difference between the two groups as regards other socio-demographic variables such as education, occupation, religion or rural/urban background. Therefore, we can conclude that the group that was analyzed was fairly representative of the population at study (*N*=103). We were not able to find any patient where there was an evidence of substituted addiction.

**Table 1:** Clinical profile of patients in study groups

Main diagnostic groups		Patient details available				*P* value (independent sample's *t*-test)
			
		Yes			No	
				
		Telephonically	Case notes	Total		
Alcohol	N (%)	13 (21.3)	7 (11.5)	20 (32.78)	13 (30.95)	
	Age in years mean (SD)	35.92 (7.93)	37.57 (8.20)	36.50 (7.85)	39.62 (7.40)	0.26 df=31
Natural opioids	N (%)	3 (4.9)	1 (1.6)	4 (6.55)	3 (7.14)	
	Age in years mean (SD)	44.67 (9.50)	25	39.75 (12.52)	41.33 (4.163)	0.84 df=5
Semi/Synthetic opioids	N (%)	17 (27.9)	11 (18.0)	28 (45.90)	21 (50.00)	
	Age in years mean (SD)	25.59 (4.82)	24.91 (3.85)	25.32 (4.40)	27.62(4.75)	0.08 df=47
Mixed	N (%)	7 (11.5)	2 (3.3)	9 (14.75)	5 (11.90)	
	Age in years mean (SD)	28.86 (6.79)	31.50 (2.12)	29.44 (6.04)	26.80 (4.97)	0.42 df=12
Total	N (%)	40 (65.6)	21 (34.4)	61 (100)	42 (100)	
	Age in years mean (SD)	30.95 (8.75)	29.76 (7.97)	30.54 (8.44)	32.21 (8.25)	0.32 df=101

P significant when <0.05

**Figure 1:**
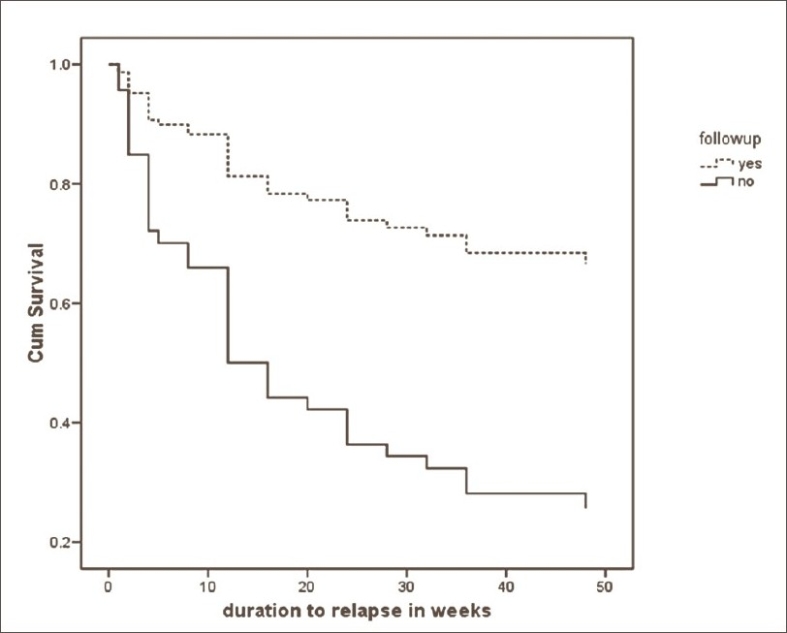
Cox's regression plots of cumulative survival times of those following-up vs. those not following-up

Table 2 shows the results of the Kaplan-Meier survival analyses with the duration to relapse as the survival time variable. The analyses reveal that the mean overall survival time was 36.35 weeks. The median survival time was 24.00 weeks (not shown). It is of interest to note that follow-up status was the only variable that seemed to exert a significant effect on survival times. Patients still following up had significantly longer survival times than those who were not. None of the other variables such as preferred substance, mode of discharge or pharmacoprophylaxis status made any statistical difference to the survival times. Therefore, follow-up status was entered into a Cox's regression analysis as a covariate using duration to relapse in weeks and abstinent and relapsed status as survival time variable and the survival status variable, respectively. Length of follow-up was controlled for in this analysis. Using this analysis, a hazard ratio of 0.29 (0.12-0.73 95% CI) was arrived at. In other words, those still following up were about 70% (27%-88% CI) more likely to be abstinent than those who were not. This is represented in the Cox's Regression plots in [Figure 1] where the cumulative survival functions of those following-up vs. those not following-up are shown.

**Table 2:** Survival times of patients with regards to variables of interest

Variables	Details of variables (*N*)	Mean survival time (Weeks)	95% CI	Log rank test
Type (*N*)	Alcohol (20)	36.71	26.24-47.18	Chi-square=4.49, df=3, *P*=0.21
	Natural Opioids (4)	34.00	8.37-59.36	
	Synthetic Opioids (28)	37.53	26.33-48.73	
	Other/Mixed (9)	17.00	3.39-30.60	
Outcome (*N*)	Improved (49)	31.45	24.52- 38.38	Chi-square=0.042, df=1, *P*=0.83
	Not improved (12)	38.00	21.26-54.74	
Pharmacoprophylaxis (*N*)	None (9)	30.22	14.97-45.46	Chi-square= 0.36, df=3, *P*=0.94
	Disulfiram (18)	28.16	18.79-37.53	
	Naltrexone (31)	36.42	25.64-47.19	
	DSF+NTX (3)	40.33	8.86-71.80	
Follow-up (N)	Yes (21)	47.29	37.24-57.34	Chi-square=8.84, df=1, *P*=0.003
	No (40)	28.06	19.58-36.54	
Overall	61	36.35	28.74-43.95	
Cox's Regression analysis of survival time with follow-up status as the covariate while			0.12-0.73	Hazard ratio=0.29, df=1, *P*=0.009
controlling for length of follow-up				

P significant when <0.05

## DISCUSSION

This study underscores the difficulties that professionals face when managing patients with SUD. These problems are manifold as patients with SUD tend to have a relapsing course and attrition rates in treatment programs are high. In our sample as well, at evaluation the number of patients who had dropped out of follow-up was about double of those who were still following-up.

However, we believe that some instructive lessons can be drawn from this study. Firstly, a majority of patients are able to maintain abstinence for about 6-9 months after in-patient treatment notwithstanding their mode of discharge. This in itself is heartening as it should discourage nihilism in the health care professionals and that in-patient treatment offers the patient and the family members some respite and harm-reduction from the effects of the substance use. Secondly, pharmacoprophylaxis may be a useful adjunct to the process of abstinence but is certainly not the only factor. In scenarios where the prophylactic agent is either unaffordable or is refused due to personal or health reasons, the prognosis is not necessarily poor. The third is the important role that follow-up has in outcomes of patients with SUD. We believe that the results of this study emphasize the role of personnel such as psychiatric social workers among others in ensuring that the patient is in regular follow-up. A look at [Table 2] shows that the mean survival times with different classes of substances were similar. While our sample size was too small to enable us to statistically compare different individual substances, it may indicate a commonality to the course of the SUD.

This was a naturalistic study in a clinical setting and thus there are some limitations to be kept in mind. No tests were carried out to objectively prove or disprove patient or attendant reports of drug use. The possibility of falsification of reporting for any reason cannot be discounted. With a view to keeping the sample homogeneous, the sample size had to be kept small. This also necessitated that explanatory variables such as individual substances, coping styles or severity of addiction[19] were not analyzed. We did not take into account lapses and occasional use of substance which is known to lead to eventual relapse in some patients.[20,21]

This study underlines the urgent need to have more methodologically sound investigations into treatment effectiveness for SUD in India.
